# Supplementary material for: First Morphological and Molecular Identification of Demodex injai in Golden Jackal (Canis aureus Linnaeus, 1758) in Romania
Source: Pathogens. 2023 Mar 5;12(3):412. doi: 10.3390/pathogens12030412 (PMC10056514; doi:10.3390/pathogens12030412)
Supplement: Supplementary file 1 [file pathogens-12-00412-s001.zip › pathogens-2236688-supplementary.pdf]

Supplementary Materials

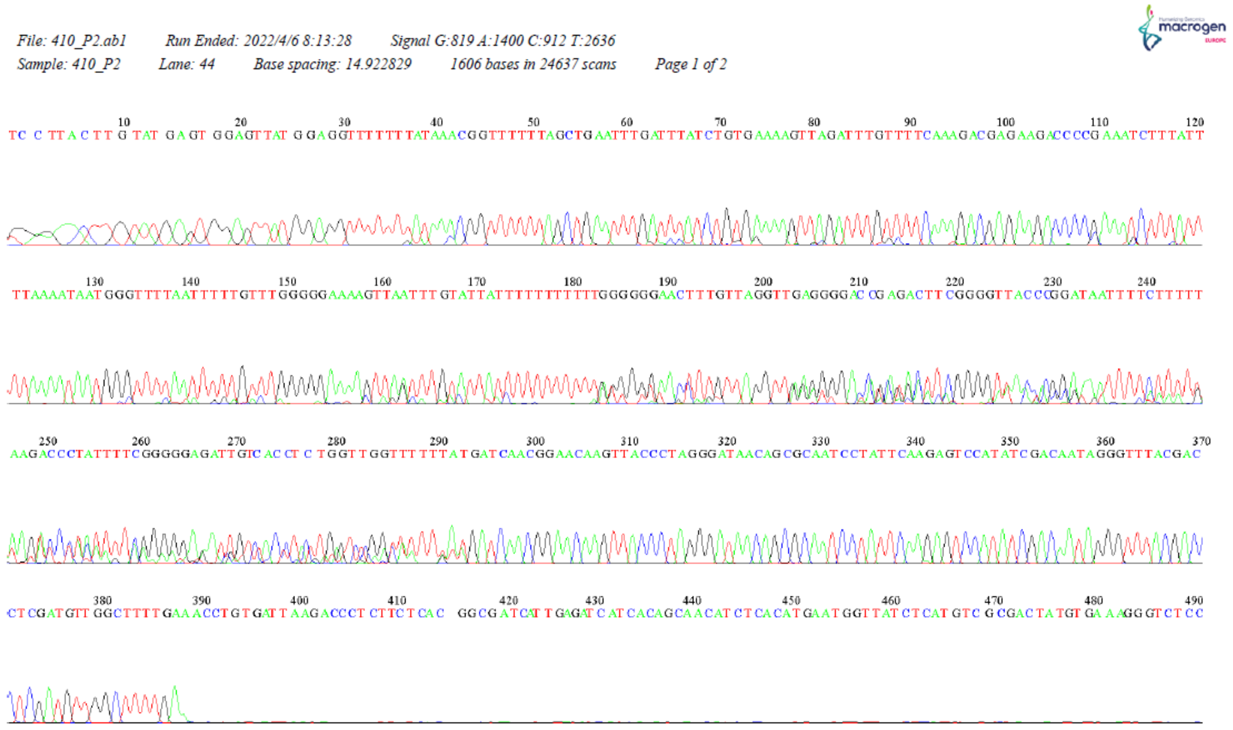

Figure S1. The sequences of the 16S rRNA gene obtained from sample 1 were identical to the *Demodex injai* sequence (GenBank nr. HE817765.1)

Nucleotide

GenBank

Demodex injai mitochondrial partial 16S rRNA gene, strain Di 1

GenBank: HE817765.1

FASTA Graphics

Go to:

```
LOCUS       HE817765                 439 bp    DNA     linear   INV 18-SEP-2012
DEFINITION  Demodex injai mitochondrial partial 16S rRNA gene, strain Di 1.
ACCESSION   HE817765
VERSION     HE817765.1
KEYWORDS    .
SOURCE      mitochondrion Demodex injai
  ORGANISM  Demodex injai
            Eukaryota; Metazoa; Ecdysozoa; Arthropoda; Chelicerata; Arachnida;
            Acari; Acariformes; Trombidiformes; Prostigmata; Eleutherengona;
            Raphignathae; Cheyletoidea; Demodicidae; Demodex.
REFERENCE   1
  AUTHORS   de Rojas,M., Riazzo,C., Callejon,R., Guevara,D. and Cutillas,C.
  TITLE     Molecular study on three morphotypes of Demodex mites (Acarina:
            Demodicidae) from dogs
  JOURNAL   Parasitol. Res. 111 (5), 2165-2172 (2012)
   PUBMED   22895570
REFERENCE   2  (bases 1 to 439)
  AUTHORS   De Rojas,M.
  TITLE     Direct Submission
  JOURNAL   Submitted (14-MAY-2012) Universidad de Sevilla, Microbiology and
            Parasitology, Profesor Garcia Gonzalez no 2, Sevilla 41012, SPAIN
FEATURES             Location/Qualifiers
     source          1..439
                     /organism="Demodex injai"
                     /organelle="mitochondrion"
                     /mol_type="genomic DNA"
                     /strain="Di 1"
                     /db_xref="taxon:1191121"
     gene            <1..>439
                     /gene="16S rRNA"
     rRNA            <1..>439
                     /gene="16S rRNA"
                     /product="16S ribosomal RNA"
ORIGIN
        1 gctcaatgat tttttaaatt gctgcggtat tttgactgtg ctaaggtagc gaagtcattt
       61 gctcttttat tgagaacttg tatgagtgga gttatggagg ttttttttata aacggttttt
      121 tagctgaatt tgatttatct gtgaaaagtt agatttgttt tcaaagacga gaagaccccg
      181 aaatctttat tttaaaataa tgggttttaa tttttgtttg ggggaaaagt taatttgtat
      241 tattttttttt tttagtgtga actttgttag gttaaatgga tagatacttc ggggttaaca
      301 ggataatttt cttttgaagt tcttatttta gagggagatt gttacctcga tgttggtttt
      361 tagtattaat tgggggtagg ttttcatatt tatagtctgt tcgactattg aatctaaaca
      421 tgatctgagt tcagaccgg
//
```

Figure S2. Sequences with the highest similarity to the PCR product obtained from the amplification of samples
